# Causal discovery replicates symptomatic and functional interrelations of posttraumatic stress across five patient populations

**DOI:** 10.3389/fpsyt.2022.1018111

**Published:** 2023-01-26

**Authors:** Benjamin Pierce, Thomas Kirsh, Adam R. Ferguson, Thomas C. Neylan, Sisi Ma, Erich Kummerfeld, Beth E. Cohen, Jessica L. Nielson

**Affiliations:** ^1^Department of Psychiatry and Behavioral Sciences, University of Minnesota, Minneapolis, MN, United States; ^2^Institute for Health Informatics, University of Minnesota, Minneapolis, MN, United States; ^3^Department of Neurological Surgery, Weill Institute for Neurosciences, Brain and Spinal Injury Center, University of California, San Francisco, San Francisco, CA, United States; ^4^San Francisco Veterans Affairs Medical Center, San Francisco, CA, United States; ^5^Department of Psychiatry and Neurology, University of California, San Francisco, San Francisco, CA, United States; ^6^Department of Medicine, University of Minnesota, Minneapolis, MN, United States; ^7^Department of Medicine, University of California, San Francisco, San Francisco, CA, United States

**Keywords:** post-traumatic stress disorder, depression, substance abuse disorders, longitudinal, machine learning, comorbidity, replication

## Abstract

**Introduction:**

Approximately half of individuals with posttraumatic stress disorder (PTSD) may meet criteria for other psychiatric disorders, and PTSD symptoms are associated with diminished health and psychosocial functioning. However, few studies examine the longitudinal progression of PTSD symptoms concurrent with related symptom domains and functional outcomes, such that may neglect important longitudinal patterns of symptom progression beyond PTSD specifically.

**Methods:**

Therefore, we used longitudinal causal discovery analysis to examine the longitudinal interrelations among PTSD symptoms, depressive symptoms, substance abuse, and various other domains of functioning in five longitudinal cohorts representing veterans (*n* = 241), civilians seeking treatment for anxiety disorders (*n* = 79), civilian women seeking treatment for post-traumatic stress and substance abuse (*n* = 116), active duty military members assessed 0–90 days following TBI (*n* = 243), and civilians with a history of TBI (*n* = 43).

**Results:**

The analyses revealed consistent, directed associations from PTSD symptoms to depressive symptoms, independent longitudinal trajectories of substance use problems, and cascading indirect relations from PTSD symptoms to social functioning through depression as well as direct relations from PTSD symptoms to TBI outcomes.

**Discussion:**

Our findings suggest PTSD symptoms primarily drive depressive symptoms over time, tend to show independence from substance use symptoms, and may cascade into impairment in other domains. The results have implications for refining conceptualization of PTSD co-morbidity and can inform prognostic and treatment hypotheses about individuals experiencing PTSD symptoms along with co-occurring distress or impairment.

## 1. Introduction

Post-traumatic stress disorder (PTSD) is highly co-morbid with internalizing conditions, including major depression and anxiety disorders. Prevalence estimates suggest over half of PTSD patients meet criteria for one such disorder (1–8). Patients with comorbid PTSD and depression have more suicide attempts and a twofold increase in medical costs, compared with either disorder alone (9–16). Similarly, comorbid anxiety disorders are associated with more severe PTSD and impairment (7, 9), often emerging in triple comorbidity with PTSD and depression (9, 17). There is debate about interpreting comorbidity among PTSD and other internalizing disorders (18), including whether PTSD symptoms drive internalizing symptoms, or vice versa, or if the relationship is bidirectional (19). Studying the connections between these symptom domains and their impacts on patients’ health and functioning can support more effective, targeted care.

Elucidating the relationship between PTSD and other internalizing conditions has been challenging and hampered by attempts to isolate symptoms of each disorder (20). Many clinical studies of PTSD exclude patients with internalizing comorbidities, or treat such symptoms as confounded, while studies of internalizing disorders often exclude patients with PTSD, or neglect to evaluate traumatic history (20). Cross-sectional studies have identified shared features of PTSD and other internalizing concerns (21–25), including network analyses using an adult cohort with depressive symptoms and trauma history, which found that impaired concentration, sleep problems, irritability, and guilt, were shared across disorders (26). Other cross-sectional analyses suggest a negative affectivity factor explains covariance across PTSD, generalized anxiety, and major depression (27, 28), and indicate common genetic and environmental correlates of these concerns (29).

Longitudinal studies including internalizing and PTSD symptoms have produced inconsistent results (30, 31). A study of combat veterans and prisoners of war assessed for depression and PTSD over three occasions found bidirectional relationships between PTSD and depressive symptoms, suggesting a common posttraumatic construct (31). In contrast, a study of urban US civilians assessed over three occasions found PTSD and depression were related bi-directionally, but were distinct constructs (30). Other research identified gender differences in directionality, finding a bi-directional relationship among women, but unidirectional association from PTSD to depression among men (19). Another community-based study found weekly changes in PTSD over a 6 to 12-week period were predicted by pre-treatment anxiety sensitivity (32). Finally, longitudinal studies of war veterans and lung injury survivors suggest anxiety, depressive, and PTSD symptoms emerge as concurrent co-morbidities (9, 33). Therefore, the current study aims to address these discrepancies by demonstrating a consistent and conserved directionality to these symptom domains.

Recent advances in machine-learning support more flexible modeling of longitudinal associations between PTSD and internalizing symptoms. Causal inference algorithms can identify the most plausible network of directional associations, supporting data-driven investigations into the relations among these symptom domains (34–36). Unlike prior studies, these algorithms can also incorporate other outcomes that could mediate relations between PTSD and internalizing symptoms, such as alcohol use or functional status. Understanding whether and how PTSD and internalizing symptoms drive health and functioning is also important.

The present study used advanced machine learning methods to investigate the directional associations among posttraumatic and internalizing symptoms along with multiple psychosocial outcomes of clinical significance. We aimed to assess the driving roles of each symptom domain more precisely through applying causal discovery techniques to longitudinal data assessing PTSD, depressive symptoms, and psychosocial outcomes over 8 years of data. We validated our findings using multiple measures of internalizing symptoms in two additional samples from longitudinal studies with distinct age, gender representation, prevalent diagnoses, and treatment settings.

## 2. Materials and methods

### 2.1. Overview

The current study focused on interrelations among post-traumatic stress and a breadth of symptom measures using data-driven computational experiments on complete longitudinal clinical data from five studies of distinct populations (Study 1, *n* = 241; Study 2, *n* = 79; Study 3, *n* = 116; Study 4, *n* = 243; Study 5, *n* = 43). Study 1 included complete longitudinal data from 8 consecutive years of the Mind Your Heart study [MYH; (37)] and including measures across domains of PTSD, depression, social functioning, physical health, and alcohol use. Study 2 included 4-wave longitudinal data across baseline, 6-month, 12-month, and 18-month follow-ups of participants with a PTSD diagnosis enrolled in a randomized controlled trail of the Coordinated Anxiety Learning and Management [CALM; (38)] intervention. Study 3 included 5-wave longitudinal data across baseline, post-intervention (6 weeks after baseline), 3-month follow-up, 6-month follow-up, and 12-month assessments from women enrolled in a randomized trial of the Seeking Safety intervention for women with post-traumatic stress symptoms and substance abuse, in the Women’s Treatment for Trauma and Substance Use Disorders study [WTTS; (39)]. Study 4 included two-wave longitudinal data from active military service members with and without concussive TBI, assessed at a baseline time-point (immediately post-TBI) then again between 6 and 24 months after baseline (ADAPT; also called “CENC Study 25;” doi: 10.23718/FITBIR/1504245). Study 5 included two-wave longitudinal data from participants with a history of TBI within the past 15 years, collected at a baseline time-point and again between 6 and 12 months after baseline (TEAM-TBI; doi: 10.23718/FITBIR/1518871).

### 2.2. Study populations

The prospective MYH Study was designed to examine associations between PTSD and health outcomes. Participants were recruited between 2008 to 2010 from the San Francisco and Palo Alto Department of Veterans Affairs (VA) medical centers. Flyers at VA facilities and mailings were used to recruit patients with and without PTSD diagnoses in the previous 5 years (40). Participants were excluded if they had a myocardial infarction 6-month prior, could not walk one block on a treadmill, did not have stable contact information, or planned to move within 3 years. The study was approved by the institutional review boards of the University of California, San Francisco and the San Francisco Veterans Administration and all participants provided written informed consent. A total of 747 enrolled participants completed baseline examinations. Participants completed annual telephone interviews with validated assessments of several health and function domains. The present study included data from 241 participants that completed assessments across 8 years.

The CALM study is a randomized-controlled trial of the CALM Tools for Living intervention for anxiety disorders in primary care (41). Participants were recruited from and received treatment at four U.S. primary care sites between 2006 and 2008. Eligibility criteria included age 18 years or older and meeting criteria for any DSM-IV anxiety disorder (further exclusion criteria reported in; 38). Participants were randomized using stratified permutated block randomization and received either the CALM intervention or treatment-as-usual. Participants assigned to the intervention chose between taking medications, the Tools for Living intervention, or both. The CALM Tools for Living intervention involved eight, hour-long, computerized cognitive-behavioral intervention modules that were supervised by an anxiety specialist clinician. A total of 1004 primary care patients who completed baseline assessments were enrolled (41), and re-assessed on outcomes at 6, 12, and 18 months post-intervention. We identified 79 participants who met diagnostic criteria for PTSD and were assessed on posttraumatic stress symptoms.

The WTTS study compared two manualized interventions for co-occurring substance abuse and posttraumatic stress among women (39). Women with substance abuse disorders and PTSD, or sub-threshold posttraumatic stress, were eligible. Participants were randomly assigned to receive either Seeking Safety or Women’s Health Education interventions to supplement ongoing treatment for substance abuse. Participants were assessed on a range of outcomes at baseline, 2-month later at post-intervention, and at 3, 6, and 12-month follow-ups. Details of the WTTS study design are published elsewhere (39). Of the 353 participants initially enrolled in the WTTS study, our analysis included 116 with complete data on all measures at each assessment point.

The ADAPT study was designed to examine clinical outcomes of active duty US military with mild TBI *via* clinical assessments and magnetic resonance imaging over a longitudinal assessment period. The study prospectively enrolled 591 active duty US military participants between 0 and 90 days post-injury, who completed longitudinal follow-up assessments between 6 and 24 months post-injury (with later follow-ups planned but not completed). Our analysis included 243 of these participants with complete 6–24 month follow-up data on clinical assessment measures.

The TEAM-TBI study enrolled 95 participants with a history of TBI in the past 15 years and administered a large battery of baseline and 6–12 month follow-up assessments to refine strategies for evaluating TBI outcomes. Of the original sample, 43 participants were included who had valid intake and 6–12 month follow-up assessments on clinical measures.

### 2.3. Measures

Measures included in each study are presented in Table 1. All studies included measures of PTSD symptoms, depressive symptoms, and alcohol/substance use concerns. The MYH, CALM, and WTTS studies additionally included assessments of participants’ perceptions of their health and physical functioning. The ADAPT and TEAM-TBI studies included assessments of post-TBI outcomes. Beyond these measures, specific assessments were also identified for inclusion based on factors unique to each study (e.g., employment problems in the WTTS study, dizziness-related impairment in the TEAM-TBI study).

**TABLE 1:** Measures included in each study sample.

Study	PTSD symptoms	Depression symptoms	Substance use problems	Post-TBI symptoms	Social functioning	Health concerns	Other domains
MYH	PTSD checklist (DSM-IV)	Patient health questionnaire	Alcohol use disorders identification test–consumption	–	36-item short-form health survey–social functioning	36-item short-form health survey–physical functioning; SF36–Gen. Health	–
CALM	PTSD checklist (DSM-IV)	Goldberg anxiety and depression scale	Alcohol use disorders identification test	–	36-item short-form health survey–social functioning	36-item short-form health survey–physical functioning; SF36–Gen. Health	–
WTTS	PTSD symptom self-report	Brief symptoms inventory	Addiction severity index–alcohol use; addiction severity index–drug use	–	–	Addiction severity index–medical status	Addiction severity index–employment status
CENC-ADAPT	PTSD checklist (DSM-V)	Patient health questionnaire	Alcohol use disorders identification test (severity)	Glasgow outcome scale–extended	–	–	–
TEAM	PTSD checklist (DSM-IV)	Patient health questionnaire	Alcohol use disorders identification test (severity); drug abuse screening test (severity)	Immediate post-concussion assessment and cognitive test; rivermead post-concussion symptoms scale	–	–	Dizziness handicap inventory

MYH, Mind Your Heart study; CALM, Coordinated Anxiety Learning and Management study; WTTS, Seeking Safety study for women with post-traumatic stress symptoms and substance abuse; CENC-ADAPT, assessment of long-term outcome and disability in active-duty military prospectively examined following concussive traumatic brain injury; TEAM-TBI, targeted evaluation, action, and monitoring of traumatic brain injury study.

### 2.4. Analyses

**FIGURE 1:**
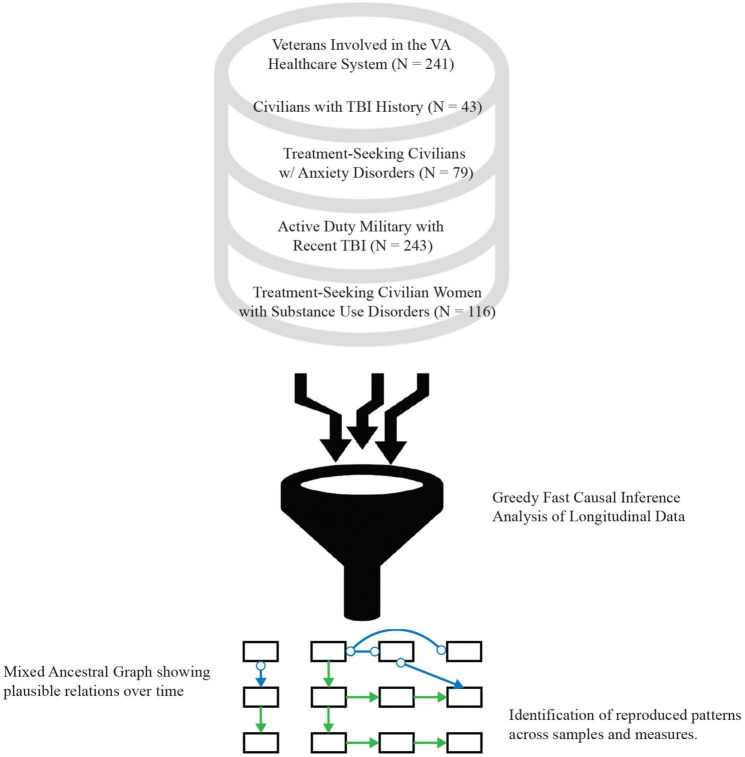
Study procedural diagram. Data from five separate patient cohorts from different clinical studies were mined for this study, spanning the range of civilians, veterans, and active-duty military populations with posttraumatic stress, substance abuse disorders, traumatic brain injury (TBI), or a combination of multiple of these as the primary diagnosis, with some compared to healthy controls.

Greedy Fast Causal Inference [GFCI; (34, 35)] analysis was performed to determine the network structure among post-traumatic stress and related outcomes in each dataset, summarized in Figure 1. GFCI uses a combination of goodness-of-fit statistics, conditional independence tests, and mathematical decision rules to search across all possible directed acyclic graphs (DAGs), including DAGs with unmeasured variables, to find the collection of DAGs most consistent with the data. DAGs are used to represent the structure of models, including Structural Equation Models [SEMs; (42)] and Bayesian Networks [BNs; (43)], and GFCI is analogous to searching through the space of all possible SEMs or BNs. This collection of DAGs is represented as a mixed ancestral graph (MAG). The lines connecting the nodes in MAGs can have a combination of different endpoints, e.g., arrowheads, arrow tails, and circles, along with different line types, capturing information about the entire set of represented DAGs.

The DAG space is extremely large and impossible to search exhaustively for the number of variables in the primary dataset. GFCI thus selects an initial set of DAGs using a depth-first search guided by artificial intelligence (AI) to optimize a comparative goodness-of-fit statistic. It then uses AI to modify these graphs to maintain consistency with conditional independence and dependence statements supported by the data. These modifications can include the addition of unobserved factors. The resulting MAG thus represents a set of DAGs which both outperform all alternative theories and are as consistent with the data as possible. This makes GFCI a robust analytic approach that maximizes information gain from highly multidimensional data without many of the shortcomings of traditional clinical prediction models.

Greedy Fast Causal Inference was run using Tetrad version 6.6.0. The primary settings and parameters were left as their defaults: Bayesian Information Criterion (BIC) score with penalty discount 2, and Fisher *Z* test with alpha 0.01. The upper bound on maximum degree was removed by setting it to −1. This was a default value for older versions of Tetrad and prioritizes accuracy over runtime. The algorithm’s search was augmented with background knowledge to restrict causal arrows pointing backward in time. Graph stability was assessed from 1,000 bootstrap samples, applying the same GFCI analysis to each, and aggregating the resulting 1,000 graphs into a table summarizing the proportion of all possible relationships. The edges in the graph were compared to those in the table to confirm that they were not regularly changed or destroyed.

#### 2.4.1. Effect size estimation

To obtain effect-size estimates, each MAG was converted to a path analysis input (44). Each directional relation was represented as a regression path, whereas non-directional relations in the MAGs were represented as covariances in the path model. Covariances among exogenous measures were allowed in the path analysis model, while covariances among endogenous measures were restricted. The resulting models thus paralleled the robust causal network represented in the MAGs, while preserving non-directional relations among variables of interest. The path models were run using the “lavaan” package in R (45) using the maximum likelihood estimation procedure with robust standard-errors. Standardized associations and confidence intervals were interpreted to obtain effect-size and uncertainty estimates associated with relations depicted in the MAGs.

## 3. Results

Table 2 shows demographic and diagnostic characteristics of participants in each sample selected for analysis for the present study. Considering demographics, participants in the MYH study were the oldest, on average, while participants in the TEAM-TBI were the youngest. Participants in MYH, ADAPT, and TEAM-TBI studies were mostly men (over 75%); participants in CALM were mostly women; and all participants enrolled in WTTS were women. Across studies, the most prevalent participant racial-ethnic identification was White and non-Hispanic/Latinx, ranging from 40.5 to 88.4% of the study samples. The next most commonly endorsed participant race-ethnicity was Black and non-Hispanic/Latinx, ranging from 4.7 to 41.4% of the study samples. The MYH, CALM, and ADAPT studies included larger proportions of participants identifying a Hispanic or Latinx ethnicity. Educational attainment also varied across studies, with the majority of MYH participants having high school degrees, the majority of CALM and TEAM-TBI participants completing some post-secondary, and most ADAPT participants having a 4-year college degree. Educational attainment was not assessed in the WTTS study. The majority of participants in all studies except WTTS reported being married, while a slight majority of WTTS participants reported they were divorced or separated. Diagnostic and TBI status similarly varied across studies, yet diagnostic information was not collected consistently. Notably, all participants in the CALM sample met criteria for both PTSD and Generalized Anxiety Disorder; all participants in WTTS met criteria for a substance use disorder; and all participants in the TEAM-TBI study had a previous TBI.

**TABLE 2:** Sociodemographics and select clinical characteristics of each study sample.

	MYH	CALM	WTTS	CENC-ADAPT	TEAM
	*n* = 241	*n* = 79	*n* = 116	*n* = 243	*n* = 43
**Demographics**					
Age	59.8 (10.3)	45.5 (12.6)	41.1 (9.13)	40.1 (10.7)	34.3 (7.0)
**Gender**					
Male	228 (94.6%)	17 (21.5%)	0 (0.00%)	213 (87.7%)	34 (79.1%)
Female	13 (5.4%)	63 (79.7%)	116 (100%)	30 (12.3%)	9 (20.9%)
Other genders	0 (0.0%)	0 (0.0%)	0 (0.0%)	0 (0.0%)	0 (0.0%)
**Race/Ethnicity**					
Asian, not Latinx or Hispanic	19 (7.9%)	1 (1.3%)	0 (0.0%)	2 (0.8%)	1 (2.3%)
Black, not Latinx or Hispanic	37 (15.4%)	15 (19.0%)	48 (41.4%)	50 (20.6%)	2 (4.7%)
White, not Latinx or Hispanic	158 (65.6%)	36 (45.6%)	47 (40.5%)	143 (58.8%)	38 (88.4%)
Another/>1 race, not Latinx or Hispanic	3 (1.2%)	12 (15.2%)	19 (16.4%)	4 (1.6%)	1 (2.3%)
Unreported race, not Latinx or Hispanic	0 (0.0%)	1 (1.3%)	0 (0.0%)	3 (1.2%)	0 (0.0%)
Latinx or Hispanic (race not reported)	14 (5.8%)	5 (6.3%)	1 (0.9%)	4 (1.6%)	1 (2.3%)
Asian and Latinx or Hispanic	1 (0.4%)	0 (0.0%)	0 (0.0%)	0 (0.0%)	0 (0.0%)
Black and Latinx or Hispanic	0 (0.0%)	0 (0.0%)	0 (0.0%)	6 (2.5%)	0 (0.0%)
White and Latinx or Hispanic	3 (1.2%)	8 (10.1%)	0 (0.0%)	28 (11.5%)	0 (0.0%)
Another/>1 race, Latinx or Hispanic	0 (0.0%)	1 (1.3%)	1 (0.9%)	0 (0.0%)	0 (0.0%)
Unreported race/Ethnicity	6 (2.5%)	0 (0.0%)	0 (0.0%)	3 (1.2%)	0 (0.0%)
**Education**					
Less than H.S	37 (15.4%)	16 (20.3%)	–	0 (0.0%)	0 (0.0%)
H.S graduate	108 (44.8%)	7 (8.9%)	–	27 (11.1%)	10 (23.2%)
Some post-secondary	52 (21.6%)	46 (58.2%)	–	95 (39.1%)	23 (53.5%)
College degree	44 (18.3%)	10 (12.7%)	–	113 (46.5%)	10 (23.2%)
Missing/Unknown	0 (0.0%)	0 (0.0%)	–	8 (3.3%)	0 (0.0%)
**Marital status**					
Never married	71 (29.5%)	13 (16.5%)	37 (31.9%)	43 (17.7%)	12 (27.9%)
Married/Common law	84 (34.9%)	35 (44.3%)	27 (23.3%)	137 (56.4%)	22 (51.2%)
Divorced/Separated	76 (31.5%)	23 (29.1%)	43 (37.1%)	59 (24.3%)	9 (20.9%)
Widowed	10 (4.1%)	3 (3.8%)	9 (7.8%)	0 (0.0%)	0 (0.0%)
Other	0 (0.0%)	5 (6.3%)	0 (0.0%)	4 (1.6%)	0 (0.0%)
**DSM diagnosis**					
Current PTSD	72 (29.9%)	79 (100%)	94 (81.0%)	–	–
12-month MDD	96 (39.8%)	66 (83.5%)	–	–	–
12-month GAD	50 (20.7%)	79 (100%)	–	–	–
Current SUD	–	–	116 (100%)	–	–
**TBI history**					
TBI history	18 (7.5%)	–	–	193 (79.4%)	43 (100%)
GOSE severe disability	–	–	–	39 (16.0%)	–
GOSE moderate disability	–	–	–	74 (30.5%)	–
RPQ score >35 (mod-severe disability)	–	–	–	–	20 (46.5%)

PTSD, post-traumatic stress disorder; MDD, major depressive disorder; GAD, Generalized Anxiety Disorder; SUD, substance use disorder; TBI, traumatic brain injury; GOSE, glasgow outcome scale–extended; RPQ, rivermead post-concussive symptoms questionnaire.

Supplementary Tables 1–5 present correlations among focal study measures included in the GFCI for each sample. Across studies, statistically significant associations were observed among measures of post-traumatic stress, depressive symptoms, TBI symptoms, and social and health functioning. Meanwhile, measures of substance abuse symptoms were not statistically significantly associated with other assessment instruments. This pattern was observed across measures of substance use problems.

### 3.1. GFCI analysis results

**FIGURE 2:**
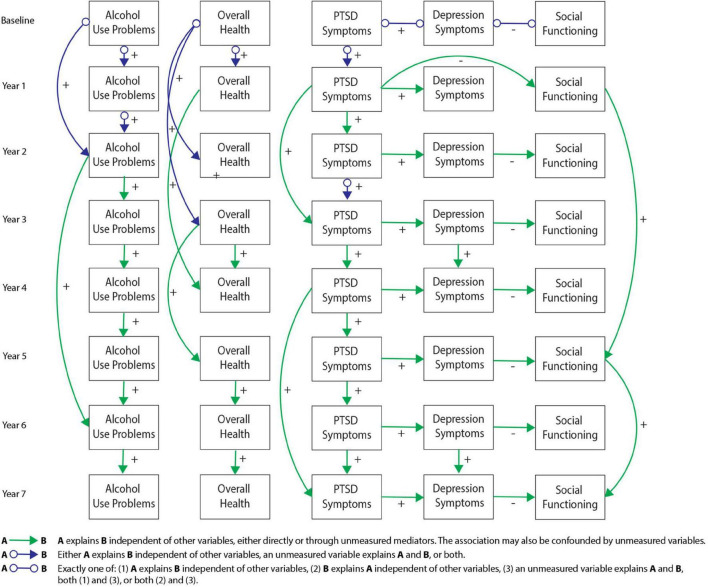
Greedy Fast Causal Inference (GFCI) graph for the Mind Your Heart study (MYH) sample (*n* = 241). Causal links between post-traumatic stress disorder (PTSD) symptoms and depression over the course of 8 years in the MYH sample, leading to social functioning, which are all independent of overall health and alcohol use problems. Colored arrows indicate the level and directionality of the causal edges, with + or – along the edges indicating whether these links cause increases or decreases in scores on the connected measures.

**FIGURE 3:**
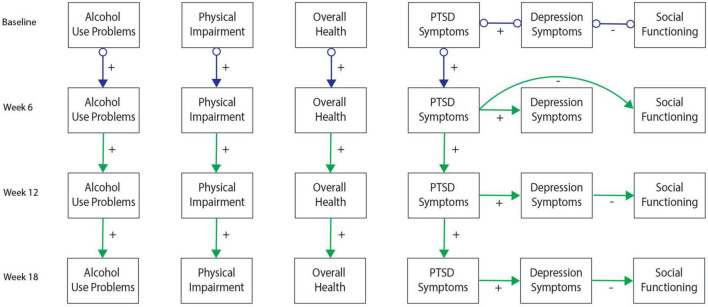
Greedy Fast Causal Inference (GFCI) graph for the Coordinated Anxiety Learning and Management (CALM) sample (*n* = 79). First independent replication of causal links between post-traumatic stress disorder (PTSD) symptoms and depression over the course of 18 weeks in the CALM sample, leading to social functioning, which are all independent of overall health, physical health, and alcohol use problems. Colored arrows indicate the level and directionality of the causal edges, with + or – along the edges indicating whether these links cause increases or decreases in scores on the connected measures.

**FIGURE 4:**
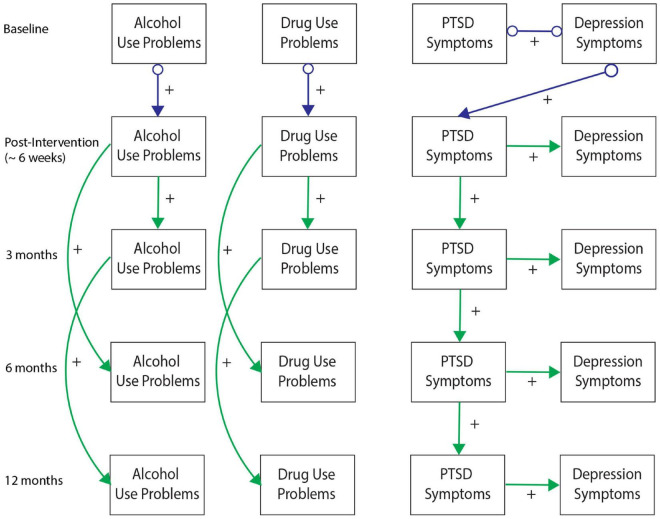
Greedy Fast Causal Inference (GFCI) graph for the WTTS sample (*n* = 116). Second independent replication of causal links between post-traumatic stress disorder (PTSD) symptoms and depression over the course of 12 months in the WTTS sample, which are both independent of drug and alcohol use problems. Colored arrows indicate the level and directionality of the causal edges, with + or – along the edges indicating whether these links cause increases or decreases in scores on the connected measures.

**FIGURE 5:**
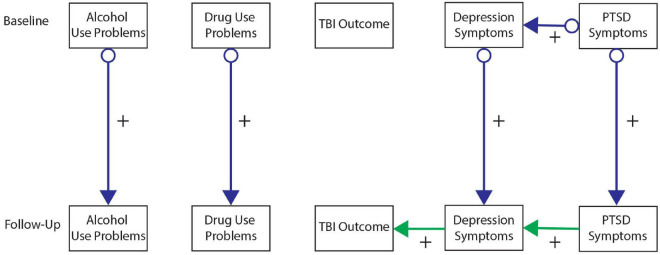
Greedy Fast Causal Inference (GFCI) graph for the CENC-ADAPT sample (*n* = 243). Third independent replication of causal links between post-traumatic stress disorder (PTSD) symptoms and depression over the course of 6–24 months follow-up in the CENC-ADAPT sample, leading to severity of traumatic brain injury (TBI) outcome, which are all independent of drug and alcohol use problems. Colored arrows indicate the level and directionality of the causal edges, with + or – along the edges indicating whether these links cause increases or decreases in scores on the connected measures.

**FIGURE 6:**
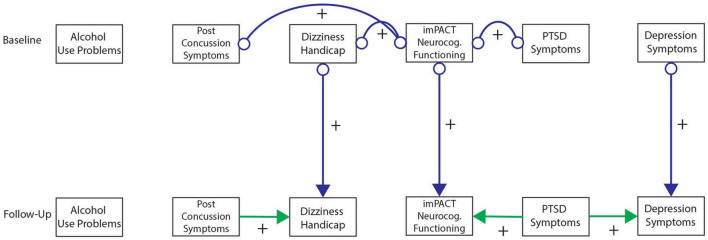
Greedy Fast Causal Inference (GFCI) graph for the TEAM-TBI sample (*n* = 43). Fourth independent replication of causal links between post-traumatic stress disorder (PTSD) symptoms and depression over the course of 6–12 months follow-up in the TEAM-TBI sample, leading to neurocognitive dysfunction, which are all independent of alcohol use problems. Colored arrows indicate the level and directionality of the causal edges, with + or – along the edges indicating whether these links cause increases or decreases in scores on the connected measures.

Figures 2–6 present patterns of associations among selected PTSD symptom and related assessments identified *via* GFCI in each study, and which showed stability across the bootstrapped analyses. Across studies, directed edges were observed from PTSD symptoms to depressive symptoms, while neither directed nor undirected edges were observed between PTSD symptoms and measures of substance use problems. As such, longitudinal variability in depressive symptoms was explained by PTSD symptoms, and this directional association was preserved independent of the potential associations between depressive symptoms and other measures. Meanwhile, longitudinal variability in substance use measures was only independently explained by prior values on those measures, such that potential associations between substance use problems and other measures added no explanatory value independent of the associations between prior and subsequent scores on substance use measures. These edges were consistent across bootstrap resamples in all studies (see Supplementary Tables 6–10).

Additional directed edges were noteworthy across studies. PTSD symptoms tended to explain measures of social functioning through depressive symptoms in both the MYH and CALM studies. While the correlational analyses suggested PTSD symptoms were moderately to strongly negatively associated with social functioning in both studies, the present results indicated that this negative association was accounted for by depressive symptoms. Depressive symptoms thus proximally explained longitudinal variability in social functioning, such that associations of social functioning with other measures provided no further explanatory value, while PTSD symptoms distally explained longitudinal variability in social functioning through uniquely explaining depressive symptoms. Conversely, PTSD symptoms indirectly explained longitudinal variability in TBI symptoms/functioning through depression in the ADAPT study while PTSD symptoms showed a direct edge toward TBI symptoms in the TEAM-TBI study independent of depressive symptoms. While these findings generally suggest PTSD symptoms have a directed association toward TBI symptoms (either directly or indirectly through depressive symptoms), this pattern was somewhat less stable across studies and bootstrap resamples. Supplementary Table 10 includes details on the instability of this association across bootstraps in the TEAM-TBI study.

Details on other measures and edges included in the GFCI’s but not shown in Figures 2–6 are included in Supplementary Tables 6–11. These edges showed less stability across time and bootstrap resamples, or shared no edges with other domains, and therefore were not selected for presentation in the primary figures.

### 3.2. Test for Simpson’s paradox

**FIGURE 7:**
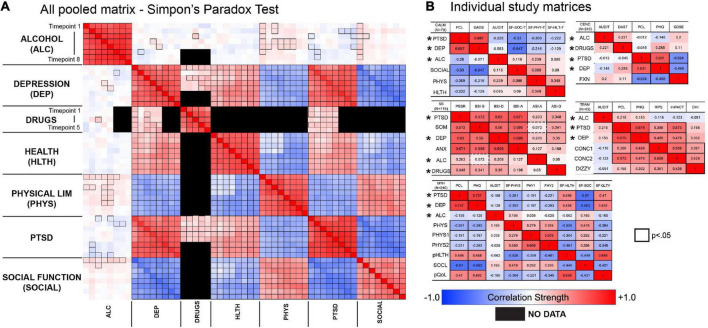
Comparison of correlation matrix from pooled sample across all five studies **(A)**, compared to individual correlation matrices for each study **(B)**. Data across all five studies were combined to test whether different relationships would emerge with the pooled sample. We found that the patterns within each dataset were mostly conserved in the pooled dataset. *Indicates common outcomes across all studies.

We pooled and normalized the data across the 5 different studies to test the Simpson’s paradox, which identifies relationships in data when pooled together that might not be represented in the individual studies themselves (46). We normalized each variable by calculating a z-score within each study for each domain that was measured. Given that different depression and substance use questionnaires were used across the different studies, this was necessary so that the variance in the pooled matrix is not explained by this scaling discrepancy (Figure 7). We see in the pooled matrix (Figure 7A) that alcohol use is weekly correlated with the other outcomes, with more correlations to PTSD and depression, however, these are not strong correlations that the other measures in the matrix have with each other, and confirms the lack of strong correlations in each individual study (Figure 7B).

## 4. Discussion

This is the first study to use a data-driven approach to identify and replicate a unidirectional relationship from PTSD to internalizing symptoms across multiple, distinct samples with varying diagnostic and demographic characteristics. Our findings provide insight into trajectories of patients with co-morbid diagnoses, with implications for treating internalizing symptoms in the context of PTSD. These results highlight the importance of measuring trauma exposure and presence of PTSD in clinical research studies of internalizing disorders and controlled clinical trials for internalizing distress.

Studies evaluating associations of PTSD and internalizing symptoms over time have had conflicting results (9, 17, 27–30). This research has found unidirectional relations between PTSD and internalizing symptoms [though directionality was not consistent; (17, 29)]; bi-directional associations (9, 27, 28, 30); and differential associations by population characteristics (e.g., gender) (17). We expand on this work by using machine learning methods that offer more flexible analyses of the associations among PTSD and internalizing symptoms, along with potential confounders. Previous research relies on testing competing *a priori* hypotheses for these relations; for instance, by specifying competing models and then assessing their correspondence to the data (27, 28). This research is often limited to a single sample with few assessment points across which to establish conserved symptom relations (19–23, 27–29). In contrast, the present study used a data-driven approach that allowed for nuanced relations to emerge across multiple timepoints in a hypothesis generation stage, and then reproduced across two additional samples. Our finding of a unidirectional association with PTSD explaining concurrent internalizing symptoms, after accounting for prior internalizing symptoms, was consistent across three cohorts representing separate studies of primarily men or women, and observational and clinical trial samples across both veteran and civilian cohorts. Beyond these results, our mechanistic analyses suggested a bridging role of hyperarousal and cognitive and affective disruption between PTSD and depressive concerns more specifically.

Our findings raise questions about the interpretation of internalizing symptoms that emerge in the context of PTSD. Such symptoms may not be accurately characterized as involving co-morbidity among distinct conditions. Instead, our results suggest these symptoms represent a downstream outcome of PTSD, which may be addressed through treating features bridging between posttraumatic stress and internalizing concerns. Consistent with our exploratory analysis of potential mechanisms, a study of Israeli veterans suggested centrality of the DSM-V negative alterations in cognitions and mood (NACM) symptom cluster in bridging among PTSD, depressive symptoms, and moral injury (25). Similarly, consistent with our findings on the centrality of hyperarousal across PTSD and depressive symptom groups, other research suggests anxiety sensitivity and avoidance of inner experiences broadly may be transdiagnostic processes underlying PTSD and internalizing symptomatology (32, 47, 48). Further longitudinal research should consider the mechanisms connecting hyperarousal, NACM, and other internalizing symptoms that could play a transdiagnostic role in comorbidities.

We also report findings that use data collected under the DSM-IV, despite the DSM-V being available for these assessments. This is not an unusual problem with longitudinal data mining studies in the PTSD field given the evolving nature of the diagnostic criteria. In general, both instruments have strong agreement in determining if someone has low, medium, high symptoms. Previous efforts to produce an equipercentile manipulation with loglinear smoothing to compute a “crosswalk” between PCL-IV and PCL-V scores has been done and are in strong agreement with measuring the range of symptom severity (49).

Our findings affirm the need to assess for posttraumatic stress in the context of research on anxious and depressive disorders. This appears especially pressing for clinical trials, as there is a lack of information about the co-occurrence of PTSD in much of this research (18). For example, we were unable to find a study with a focus on internalizing symptoms and sufficient sample size that also assessed for PTSD across the NDA and NIDA repositories. This issue may be less a result of actively excluding patients with co-morbidities, but more a product of limited assessments of co-morbid symptomology in such studies (50). Conversely, studies may exclude more severe or sub-threshold manifestations of either PTSD or internalizing symptoms (51), inadvertently restricting information about possible co-morbid conditions. A lack of information about co-morbidity may drive inaccurate care when a trauma-informed approach is not taken, and may neglect crucial mechanisms that could support clients’ recovery.

While our primary analyses concerned the relation between PTSD and internalizing symptoms, our exploratory analyses generated other hypotheses pertaining to PTSD and psychosocial outcomes. Specifically, our GFCI analyses on the MYH cohort suggested social functioning is indirectly affected by PTSD symptoms through depressive symptoms. This finding aligns with studies showing that treating interpersonal problems in PTSD can ameliorate comorbid major depression (52, 53). Conversely, our findings suggest changes in PTSD symptoms provide little information about changes in alcohol use, physical functioning, and overall health over time, and vice versa. Individuals with alcohol use or physical health concerns may thus benefit from combined treatment approaches. The alcohol use findings are noteworthy as previous studies have documented co-morbidities among PTSD and alcohol abuse, yet rarely examine whether PTSD symptoms drive alcohol abuse longitudinally (54).

Our finding on the independence of substance abuse from PTSD and depression underscores the importance of other drivers of substance use that are outside the realm of the “self-medication hypothesis.” Koob and Volkow stressed the importance of reward circuitry, which suggests that people use substances to seek out pleasurable reward sensations, and not so much to self-medicate mood/anxiety states (55). This could also be further explained regarding current, rather than past substance use, as is the case with the AUDIT. Clinical experience provides insights about this in the treatment of patients with PTSD that have a history of substance use disorder, but after going through treatment, are now actively abstaining from using alcohol, and therefore the AUDIT may not be picking up on their historical use and the association of chronic PTSD symptoms that have evolved if most of them are not endorsing current use. From a clinical perspective, there is a fair amount of literature describing the co-occurrence of diagnosed PTSD and alcohol or substance use disorders, but fewer studies looking at longitudinal relations among symptoms of those disorders specifically. The nature of the longitudinal relationships identified in our findings and continuous self-report measures may differ from those documented in cross-sectional studies with categorically defined diagnostic interviews. From a measurement standpoint, one of the caveats of the alcohol use measures included in these studies is that they are asking about drinking problems in general, versus drinking specifically to cope with post-traumatic stress symptoms or to avoid/suppress other inner experiences (54). Given that, our findings are important to replicate in future research integrating PTSD symptoms and psychosocial outcomes.

Our results are subject to several limitations. While GFCI can infer “causality” from repeated measures based on statistical criteria, the MYH study was observational. Typically, causality is confirmed with data from randomized controlled trials. We cannot randomize patients to develop psychiatric disorders, but machine learning techniques can be applied to repeated measures of PTSD, internalizing symptoms, and psychosocial outcomes collected during intervention trials to overcome this. Our validation cohorts were drawn from clinical trials, but only a subset of participants had both PTSD and internalizing symptoms assessed. Therefore, replication with thorough measurement of both symptom domains will be important. Second, though we conducted our analyses in samples of largely male veterans, women with substance use disorders, and a subset of patients enrolled in an anxiety trial, validation in populations with diversity in other experiences and identities is crucial. Third, patients were excluded due to missing data on repeated assessments, although findings based on fewer time-points and excluding fewer patients were consistent with those on all time-points. Finally, our samples largely represented patients with PTSD, so replication with other salient clinical concerns would be valuable.

Despite these limitations, our finding that PTSD symptoms drove internalizing symptoms in three separate studies suggests an etiological connection from trauma symptomology to internalizing distress. This supports prior work suggesting evolution of other internalizing symptoms secondary to PTSD could represent a manifestation of the original response to traumatic stress (7, 9, 28). Our results underscore the need for integrated therapies that treat mechanisms underlying a constellation of patients’ symptoms, rather than addressing single disorders. Finally, this work highlights the importance of longitudinal data incorporating simultaneous assessment of PTSD, internalizing distress, and patient-centered outcomes in both observational and trial settings.

## Data availability statement

The data analyzed in this study is subject to the following licenses/restrictions: Data are available to access from federal repositories managed by the National Institutes of Mental Health (NIMH), Drug Abuse (NIDA), and Neurological Disorders and Stroke (NINDS). Data from clinical trials funded by these agencies are available to qualified researchers by setting up a data use agreement (DUA/DUC) which requires affiliation with an institution with a federal wide assurance (FWA). Data and/or research tools used in the preparation of this manuscript were obtained from the National Institute of Mental Health (NIMH) Data Archive (NDA). NDA is a collaborative informatics system created by the National Institutes of Health to provide a national resource to support and accelerate research in mental health. Dataset identifier(s): Coordinated Anxiety Learning and Management (CALM) study (Study 2146 in NDA). This manuscript reflects the views of the authors and may not reflect the opinions or views of the NIH or of the Submitters submitting original data to NDA. Data and/or research tools used in the preparation of this manuscript were obtained and analyzed from the controlled access datasets distributed from the Department of Defense (DOD)- and NIH-supported Federal Interagency Traumatic Brain Injury Research (FITBIR) Informatics Systems. FITBIR is a collaborative biomedical informatics system created by the Department of Defense and the National Institutes of Health to provide a national resource to support and accelerate research in TBI. Dataset identifier(s): CENC Study 25: Assessment of long term outcome and disability in active-duty military prospectively examined following concussive TBI: the ADAPT study (doi: 10.23718/FITBIR/1504245), and Targeted evaluation, action, and monitoring of TBI (doi: 10.23718/FITBIR/1518871). This manuscript reflects the views of the authors and may not reflect the opinions or views of the DOD, NIH, or of the Submitters submitting original data to FITBIR Informatics System. The information reported here results from secondary analyses of data from clinical trials conducted by the National Institute on Drug Abuse (NIDA). Specifically, data from Women’s Treatment for Trauma and Substance Use Disorders study (Study NIDA-CTN-0015) were included. NIDA databases and information are available at (https://datashare.nida.nih.gov). Additional data from the Mind Your Heart study is available upon request from the Principal Investigator, BC (https://mindyourheartstudy.ucsf.edu/contact-us). Requests to access NDA, FITBIR, and NIDA Data Share datasets should be directed to https://nda.nih.gov/; https://fitbir.nih.gov/; and https://datashare.nida.nih.gov/.

## Ethics statement

The studies involving human participants were reviewed and approved by University of Minnesota Institutional Review Board, STUDY00003370. Written informed consent for participation was not required for this study in accordance with the national legislation and the institutional requirements.

## Author contributions

JN, EK, SM, AF, TN, and BC conceived of the study. BC and TN collected or oversaw collection of MYH primary data. JN, TK, and BP performed data query and wrangling of MYH and NDA datasets. EK performed GFCI on datasets. JN, EK, BP, SM, AF, TN, and BC were involved in interpreting results. JN, EK, SM, TK, BP, AF, TN, and BC wrote the manuscript. All authors contributed to the article and approved the submitted version.
