# Supplementary material for: Causal discovery replicates symptomatic and functional interrelations of posttraumatic stress across five patient populations
Source: Front Psychiatry. 2023 Jan 26;13:1018111. doi: 10.3389/fpsyt.2022.1018111 (PMC9924232; doi:10.3389/fpsyt.2022.1018111)
Supplement: Supplementary file 1 [file Data_Sheet_1.zip › Supplementary Material_corrected 2/Supplementary Material.docx]

Supplementary Material

# Supplementary Tables

Table S1. Mind Your Heart study baseline correlation matrix (n = 241)

|  | PCL | PHQ | AUDIT | SF-PHYS | SF-HLTH | SF-SOC |
| --- | --- | --- | --- | --- | --- | --- |
| PCL | 1 |  |  |  |  |  |
| PHQ | .737* | 1 |  |  |  |  |
| AUDIT | -.158 | -.128 | 1 |  |  |  |
| SF-PHYS | -.381* | -.393* | .166 | 1 |  |  |
| SF-HLTH | .456* | .458* | -.062 | -.526* | 1 |  |
| SF-SOC | -.670* | -.663* | .165 | .418* | -.449* | 1 |

*Note.* PCL = PTSD Checklist. PHQ = Patient Health Questionnaire. AUDIT = Alcohol Use Disorders Identification Test. SF-PHYS = Short Form Health Survey – Physical Functioning score. SF-HLTH = Short Form Health Survey – Overall Health rating. SF-SOC = Short Form Health Survey – Social Functioning rating. Correlations involving the AUDIT, SF-HLTH, and SF-SOC were estimated via Spearman’s rank-order coefficient. Other correlations estimated via Pearson’s product-moment coefficient. Correlations with an * are statistically significant at *p* < .05.

Table S2. Coordinated Anxiety Learning and Management baseline correlation matrix (n = 79)

|  | PCL | GADS | AUDIT | SF-SOC-T | SF-PHY-T | SF-HLT-T |
| --- | --- | --- | --- | --- | --- | --- |
| PCL | 1 |  |  |  |  |  |
| GADS | .687* | 1 |  |  |  |  |
| AUDIT | -.280 | -.071 | 1 |  |  |  |
| SF-SOC-T | -.530* | -.647* | .115 | 1 |  |  |
| SF-PHY-T | -.309* | -.216* | .239* | .386* | 1 |  |
| SF-HLT-T | -.222* | -.129 | .085 | .090 | .348* | 1 |

*Note.* PCL = PTSD Checklist. GADS = Goldberg Anxiety and Depression Scale. AUDIT = Alcohol Use Disorders Identification Test. SF-SOC-T = Short Form Health Survey – Social Functioning t-score. SF-PHY-T = Short Form Health Survey – Physical Functioning t-score. SF-HLT-T = Short Form Health Survey – Social Functioning t-score. Correlations involving the AUDIT were estimated via Spearman’s rank-order coefficient. Other correlations estimated via Pearson’s product-moment coefficient. Correlations with an * are statistically significant at *p* < .05.

Table S3. Women’s Treatment for Trauma and Substance Use baseline correlation matrix (n = 116)

|  | ASI-A | ASI-D | PSSR | BSI-D | ASI-M | ASI-E |
| --- | --- | --- | --- | --- | --- | --- |
| ASI-A | 1 |  |  |  |  |  |
| ASI-D | .050 | 1 |  |  |  |  |
| PSSR | .203* | .348* | 1 |  |  |  |
| BSI-D | .205* | .350* | .630* | 1 |  |  |
| ASI-M | .029 | .283* | .316* | .144 | 1 |  |
| ASI-E | .055 | -.340* | -.023 | .049 | -.024 | 1 |

*Note.* ASI-A = Addiction Severity Index, Alcohol Use problems. ASI-D = Addiction Severity Index – Drug Use problems. ASI-M = Addiction Severity Index – Medical problems. ASI-E = Addiction Severity Index – Employment problems. PSSR = PTSD Symptoms Self-Report scale. BSI-D = Brief Symptoms Inventory – Depression symptoms scale. All correlations estimated via Pearson’s product-moment coefficient. Correlations with an * are statistically significant at *p* < .05.

Table S4. ADAPT study baseline correlation matrix (n = 243)

|  | AUDIT | DAST | PCL | PHQ | GOSE |
| --- | --- | --- | --- | --- | --- |
|  | 1 |  |  |  |  |
| DAST | .221 | 1 |  |  |  |
| PCL | -.012 | -.045 | 1 |  |  |
| PHQ | -.148 | .285 | .631* | 1 |  |
| GOSE | .200 | .110 | -.524* | -.498* | 1 |

*Note.* AUDIT = Alcohol Use Disorder Identification Test severity category. DAST = Drug Abuse Screening Test severity category. PCL = PTSD Checklist. PHQ = Patient Health Questionnaire. GOSE = Glasgow Outcome Scale – Extended severity rating (higher scores indicate better recovery). Correlations estimated as Spearman’s rank-order coefficient, except for that between the PCL and PHQ which was estimated via Pearson’s product-moment correlation. Correlations with an * are statistically significant at *p* < .05.

Table S5. TEAM-TBI study baseline correlation matrix (n = 43)

|  | AUDIT | PCL | PHQ | RPQ | ImPACT | DHI |
| --- | --- | --- | --- | --- | --- | --- |
| AUDIT | 1 |  |  |  |  |  |
| PCL | .215 | 1 |  |  |  |  |
| PHQ | .153 | .679* | 1 |  |  |  |
| RPQ | -.116 | .386* | .489* | 1 |  |  |
| ImPACT | -.123 | .573* | .479* | .559* | 1 |  |
| DHI | -.091 | .198 | .302* | .361* | .429* | 1 |

*Note.* AUDIT = Alcohol Use Disorder Identification Test severity category. PCL = PTSD Checklist. PHQ = Patient Health Questionnaire. RPQ = Rivermead Post-Concussion Symptoms Questionnaire. ImPACT = Immediate Post-Concussion Assessment and Testing Protocol. DHI = Dizziness Handicap Inventory. Correlations estimated as Pearson’s correlation coefficient, except for those involving the AUDIT which were estimated via Spearman’s rank-order coefficient. Correlations with an * are statistically significant at *p* < .05.

## Supplementary Tables S6 through S11 attached as separate comma separated values (CSV) files.

Table S6. List of edges from the MYH GFCI analysis, including consistency across bootstraps, estimate, and 95% confidence interval.

Table S7. List of edges from the CALM GFCI analysis, including consistency across bootstraps, estimate, and 95% confidence interval.

Table S8. List of edges from the WTTS GFCI analysis, including consistency across bootstraps, estimate, and 95% confidence interval.

Table S9. List of edges from the CENC GFCI analysis, including consistency across bootstraps, estimate, and 95% confidence interval.

Table S10. List of edges from the TEAM-TBI GFCI analysis, including consistency across bootstraps, estimate, and 95% confidence interval.

Table S11. Variable dictionary for Tables S6 through S10.
